# Formulation of Topical Bioadhesive Gel of Aceclofenac Using 3-Level Factorial Design

**Published:** 2011

**Authors:** Sanjay Singh, Rabinarayan Parhi, Anuj Garg

**Affiliations:** *Department of Pharmaceutics, Institute of Technology, Banaras Hindu University, Varanasi, 221005, India.*

**Keywords:** Topical bioadhesive gel, Aceclofenac, Three-level factorial design, Design, HPMC

## Abstract

The objective of this work was to develop bioadhesive topical gel of Aceclofenac with the help of response-surface approach. Experiments were performed according to a 3-level factorial design to evaluate the effects of two independent variables [amount of Poloxamer 407 (PL-407 = X_1_) and hydroxypropylmethyl cellulose K100 M (HPMC = X_2_)] on the bioadhesive character of gel, rheological property of gel (consistency index), and *in-vitro *drug release. The best model was selected to fit the data. Mathematical equation was generated by Design Expert® software for the model which assists in determining the effect of independent variables. Response surface plots were also generated by the software for analyzing effect of the independent variables on the response. Quadratic model was found to be the best for all the responses. Both independent variable (X_1_ and X_2_) were found to have synergistic effect on bioadhesion (Y_1_) but the effect of HPMC was more pronounced than PL-407. Consistency index was enhanced by increasing the level of both independent variables. An antagonistic effect of both independent variables was found on cumulative percentage release of drug in 2 (Y_3_) and 8 h (Y_4_). Both independent variables approximately equally contributed the antagonistic effect on Y_3_ whereas antagonistic effect of HPMC was more pronounced than PL-407.

The effect of formulation variables on the product characteristics can be easily predicted and precisely interpreted by using a 3-level factorial experimental design and generated quadratic mathematical equations.

## Introduction

Osteoarthritis (OA) is the most prevalent disease after the age of 65 in about 60% of men and 70% of women (1). OA is primarily a non-inflammatory degenerative joint disease characterized by progressive loss of articular cartilage, subchondrial bone sclerosis, osteophyte formation, changes in the synovial membrane, and an increased volume of synovial fluid with reduced viscosity and hence, changed lubrication properties. Current treatment options for OA are limited. They include intra-articular (IA) injection of glucocorticoids and hyaluronic acid (HA) preparations or symptomatic treatment with simple non-steroidal anti-inflammatory drugs (NSAIDs) (2). Due to the localized nature of the disease, the IA injection of glucocorticoids and HA seems to be an attractive approach for OA, but they provide only short-term pain relief and/or often do not provide adequate pain relief and have no patient compliance (2). On the other hand, Rheumatoid arthritis (RA) is a common chronic systemic inflammatory disease that is primarily characterized by inflammation of the synovium with destruction of cartilage and bone as the disease progresses. More severe disease may be associated with vasculitis, pericarditis, pleural effusion, pulmonary interstitial fibrosis, peripheral neuropathies, subcutaneous and pulmonary nodules and scleritis (3, 4). Since there is no cure, symptomatic treatment is the only choice to reduce the pain and inflammation. Formulations of NSAIDs are the first choice remedies which would fulfill the need of providing significantly long remission of pain with improved patient compliance.

Aceclofenac is a potent non-steroidal anti-inflammatory drug that prevents prostaglandin synthesis by inhibiting enzyme cyclooxygenase. Aceclofenac is widely used for the treatment of rheumatoid arthritis, osteoarthritis and ankylosing spondylitis (5-7). However, the oral administration of aceclofenac has often resulted in side effects, including gastrointestinal ulcer and anemia due to gastrointestinal bleeding (6). It requires frequent oral dosing because of its short half-life (3-4 h). Topical administration of aceclofenac would be a possible alternative offering distinct advantages such as elimination of the absorption variable rate, first pass intestinal and hepatic metabolism inherent with oral dosing and delivering the drug directly to the inflamed site and thereby, producing high local concentrations and avoiding the side effects (7).

The skin is the largest organ of the body, accounting for more than 10% of body mass. It has important protective and homeostatic roles and is generally regarded as a critical protective barrier to the external environment (9). A drug molecule will encounter several diffusional resistances in the course of skin permeation. The stratum corneum (SC), the outermost layer of the epidermis, is the rate limiting membrane to percutaneous delivery (10). To be therapeutically beneficial, the barrier properties of skin must be modified in such a way that the drug can be administered at a sufficiently high rate to achieve a therapeutically effective level in the proper site. There are several approaches which can be utilized to alter the barrier properties of the skin and so the percutaneous permeation rate of drugs (11). One of the useful approaches is the co-administration of skin permeation enhancers. Nowadays, topical drug delivery is a well-accepted way of delivering drugs locally and currently topical gels are used to treat the pain and inflammation in rheumatoid arthritis, osteoarthritis and ankylosing spondylitis and also in various skin diseases.

The topical administration of bioadhesive gels has distinct advantages over the other delivery system (such as good accessibility) and can be applied for localized purposes. Because of its excellent accessibility, the self-administration of a drug is possible and can be stopped at any time (12). Some studies have shown that after the topical application, significant levels of anti-inflammatory drugs were found in deep tissues such as facia, muscle, and synovium, which is a desirable feature for the relief of local symptoms (13). Rabinowitz *et al*. (1982) found that in articular tissues and synovial fluids, topical application of salicylates results in higher drug concentrations than that of oral aspirin in dogs (14). Topical bioadhesive gels of various drugs have been developed such as flurbiprofen, benzocaine, tetracaine, ketamine, piroxicam, tretinoin and griseofulvin (13, 15-19). Literature survey indicates that topical bioadhesive thermo sensitive gel for aceclofenac has not been investigated yet.

The optimum rheological properties are required for the gel to ease the application on the skin. The high viscosity of the gel makes it difficult to apply on the skin. However, low viscosity leads to rapid loss of gel from the application site. Therefore, ideal gel should be initially in fluid state for the ease of application and after being applied on the body at 37°C, it turns into the viscous gel state and shows the bioadhesiveness (20). It has been known that poloxamer exhibits the phenomenon of reverse thermal gelation, remaining as a clear solution from 20% to 30% (w/w) at low temperature and it forms a gel on warming at room temperature by undergoing sol-gel transition (21, 22). Therefore, it was proposed to develop a topical bioadhesive gel system of aceclofenac with Poloxamer 407 (PL407) and hydroxypropyl methylcellulose (HPMC) as polymers. Poloxamer 407, a non-toxic copolymer with average molecular weight of 11500, contains 70% hydrophilic ethylene oxide units and 30% hydrophobic propylene oxide units. However, in the development of formulation containing a drug that was poorly water-soluble and solubilized in the aqueous medium by poloxamers, the bioadhesive polymers such as carbopol, polycarbophil and sodium alginate could not be used, since the drug was precipitated in the preparation (23). HPMC was used to control drug release from several pharmaceutical systems due to its non-toxic nature and swelling properties.

As a result, the aim of the present study was to formulate and evaluate the topical bioadhesive thermo-sensitive gel of aceclofenac using the combination of Poloxamer 407 and HPMC. In the development of pharmaceutical dosage form with appropriate characteristics, an important issue is to design an optimized pharmaceutical formulation in a short time of period with minimum trials. To that end, response surface methodology (RSM) is currently gaining attention to identify and quantify the effect of different formulation variables on the important characteristics. In this study, a 3-level factorial design was employed to determine the effect of independent variables such as the amount of PL-407 and HPMC on the formulation characteristics such as bioadhesion, rheological properties and *in-vitro *release studies.

## Experimental

*Material*

Aceclofenac and HPMC K100 M were generously gifted by Lupin Labs (Pune, India). 

Poloxamer 407 (Lutrol F 127) was obtained from BASF (BASF, India). All other chemicals used were of analytical grade.

*Experimental design*

A 3-level factorial design was used to study the effect of two formulation variables on characteristics of topical gel such as bioadhesiveness, viscosity and *in-vitro *release studies. The amount of Poloxamer 407 and HPMC were the two numeric factors (independent variables). The studied responses (dependent variables) were bioadhesion, consistency index (rheological property) and percentage of drug release in 8 h. Dependent and independent variables along with their levels were listed in Table 1. Experimental design for preparation of different batches of bioadhesive topical gel was summarized in Table 2. 

**Table 1:** Factors (independent variables), factor levels and responses (dependent variables) used in 3-level factorial experimental design

**Factors**	**Type of factor**	**Factor level used**			**Response**
X_1_- Amount of Poloxamer 407 in gel (%w/w)	Numeric	20	25	30	Y_1_- Bioadhesive character (in gf)
X_2_- Amount of HPMC in gel (%w/w)	Numeric	2	3	4	Y_2_- Consistency index (in dyne/cm sq)
					Y_3_- CPR in 2 h (%)
					Y4- CPR in 8 h (%)

**Table 2:** Experimental design for preparation of different batches

**Std**	**Run**	**Factors**	
		**X**_1_ **- %w/w Poloxamer 407 in gel**	**X**_2_ **- %w/w HPMC in gel**
4	1	20	3
5	2	25	3
10	3	25	3
9	4	30	4
3	5	30	2
6	6	30	3
12	7	25	3
13	8	25	3
8	9	25	4
11	10	25	3
7	11	20	4
1	12	20	2
2	13	25	2

*Preparation of HPMC-PL407 gel containing aceclofenac*

PL407 possesses reverse thermal gelling property and therefore, the gel containing PL407 was prepared by cold method (24, 25). Weighed PL407 was added slowly in cold distilled water (approximately 5°C controlled by using ice bath) with continuous stirring and HPMC was dissolved in normal distilled water and stirred, simultaneously. Then, the HPMC solution was added to PL407 solution with stirring. Accurately weighed aceclofenac was dissolved in 30 mL of ethanol and the ethanolic solution of drug was added slowly in the previously prepared polymer gel with continuous stirring at 400-600 rpm. The volume was made up to 100 g with distilled water. The prepared gel was kept for 24 h at refrigerator temperature for complete polymer desolvation.

*Evaluation of topical bioadhesive gel*

*Drug content uniformity of aceclofenac bioadhesive gels*

All prepared gels were analyzed for the desired range of aceclofenac content and the samples which come within the range of 100 ± 10 were taken for *in-vitro *release studies. A hundred mg of gel was taken in 10 mL of water and mixed for 15 min (25). After filtration, 0.5 mL of solution was diluted to 5 mL by means of water and the absorbance of the solution was measured at 275 nm spectrophotometrically (Hitachi UV-Spectrophotometer). The experiments were done in triplicate.

*Bioadhesive testing by modified arm balance method*

The bioadhesive testing has been performed using modified two arm balance methods as described in our previous work. Briefly, the weighed gel (0.5 g) was placed on the intestine glued to the upper side of the lower plate. Then, the upper plate was placed over the lower plate and 50 g preload force (or contact pressure) was applied for 5 min (preload time). After the removal of preload force, the water kept in a bottle at some height was siphoned in the container at the rate of 10 mL per min till the plates were detached from each other. The rate of water dropping was controlled with an on/off switch identical to the infusion bottle. The weight of water required for detachment of glass plates was considered as the bioadhesion force of the applied gel (26).

*Rheological studies*

The samples were placed in beaker and were allowed to equilibrate for 30 min before measuring the viscosity. Viscosity determinations of the prepared formulations were carried out by Brookfield Rheometer fitted with spindle 61 at angular velocity 5, 10 and 20 rpm at room temperature. The average of the readings was used to calculate the viscosity (27). The consistency index and flow index were calculated from the Power law equation (28).

τ = Kr^n^

Where: “τ” is shear stress; “r” is shear rate; “K” is consistency index; “n” is flow index.

*In-vitro dissolution study of Aceclofenac gels*

Accurately weighed gel (equivalent to 15 mg of the drug) was taken in the center of a hollow cylindrical dialysis membrane. This membrane was then folded and hermetically sealed from both the ends. This “dialysis bag” was then hanged with the help of a wire in a 500 mL beaker (13, 29, 30). Two hundred mL of phosphate buffer (PB) with pH of 6.8 was added to dissolution medium**. **The entire system was kept at 37°C with continuous magnetic stirring at 200 ± 5 rev/min. Sampling of 1 mL was done at predetermined time-intervals of 5, 10, 15, 30, 60, 120, 180, 240, 300, 360 and 420 min. The media volume was maintained by adding equal volumes of fresh media. The concentration of aceclofenac was spectrophotometrically measured at 275 nm. The interference studies were done in order to ascertain that the polymers do not interfere with the analysis of the drug. UV scans of 0.1% drug, 0.1% polymers and combination of polymers and drug (0.1% each) revealed that the drug exhibited λ_max_ at 275 nm in PB with pH of 6.8 and polymer showed no absorbance at the same wavelength.

The saturation-solubility (Cs) of Aceclofenac in PB with pH of 6.8 is 7.53 ± 0.01 mg/mL at 37°C. Sink conditions were maintained for release studies (C < Cs × 0.1). Therefore, final concentration of Aceclofenac after the complete release in PB with pH of 6.8 was maintained less than 75 μg/mL in compliance with the sink condition.

*Statistical analysis of the data*

Various RSM computations for the current study were performed employing 30 days Trial Version of Design-Expert software (Version 7.1.2, Stat-Ease Inc., Minneapolis, MN). Polynomial models including interaction and quadratic terms were generated for all the response variables using multiple linear regression analysis. Equations were derived and coefficients of interactions were calculated to determine the effect of each variable on the formulation characteristics. Statistical validity of the model was established on the basis of Analysis of variance (ANOVA) and the 3D response graphs were constructed using Design-Expert software.

## Results and Discussion

*Experiments of 3-level factorial design*

Response data for all the 13 experimental runs of 3-level factorial design, performed in accordance with Table 2, are presented in Table 3.

**Table 3:** Results for bioadhesion, consistency index and *in-vitro *drug release studies of prepared topical bioadhesive gel with 3-level factorial experimental design

**Run**	**Response**			
	**Y**_1_ **= bioadhesion (gf)**	**Y**_2_ **= Consistency Index** **(in dyne/cm**^2^**)**	**Y**_3 _ **= CPR at 2 ** **(h)**	**Y**_4_ **= CPR at 8 ** **(h)**
1	53	8881	27.57	53.8
2	60	10127	22.81	50.86
3	61	10154	22.81	50.86
4	92	20653	16.66	38.17
5	37	5139	21.82	53.56
6	70	13001	17.74	44.21
7	61	10092	22.97	51.14
8	59	10198	22.45	50.56
9	85	20356	19.82	44.56
10	62	10154	22.34	50.38
11	68	16368	24.19	47.95
12	15	3556	39.67	66.98
13	20	4563	29.78	60.53

*Mathematical modeling*

Mathematical relationship was generated between the factors (independent variables) and responses (dependent variables) using the statistical package Design-Expert. First step in mathematical modeling was fitting the experimental data to appropriate model. A suitable model was selected by software on the basis of different parameter obtained from regression analysis such as p-value, adjusted R^2^, predicted R^2^ and Predicted Residual Sum of Square (PRESS) value (Tables 4 and 5). Table 3 lists the values of various response parameters of the prepared batches. ANOVA was applied for estimating the significance of model, at 5% significance level. If more than one model was significant (p < 0.05) for the response, the adjusted R^2^ and PRESS value of the model were compared to select the best mathematical model for that response. Focus on maximizing the value of adjusted R^2^ and predicted R^2^. Low PRESS value indicated adequate fitting of model (31). General quadratic equation for two independent variables is as follow:

Y = *β*_0_ + *β*_1_X_1_ + *β*_2_X_2 _+ *β*_3_X_1_ X2 + *β*_4_X_1_^2 ^+ *β*_5_X_2_^2^

Where: *β*_0_ is the intercept representing the arithmetic averages of all the quantitative outcomes of 13 runs. *β*_1_ to *β*_5_ are all coefficients calculated from the observed experimental values of Y. X_1_ and X_2_ are the coded levels of factors. The terms X_1_X_2_ and X_i_^2^ (i є {1, 2}) represent the interaction and quadratic terms, respectively. Coefficients with one factor represent the effect of that particular factor while the coefficients with more than one factor and those with second order terms represent the interaction between those factors and the quadratic nature of the phenomena, respectively. Synergistic effect and antagonistic effect of factor were indicated by positive sign and negative sign in front of that factor term, respectively.

**Table 4:** Fit summary of model for the measured responses Y_1_ (bioadhesion in gf), Y_2_ (Consistency index in dyne/cm^2^), Y_3_ (Cumulative percentage release at 2 h) and Y4 (Cumulative percentage release at 8 h).

**Source**	**Y**_1_		**Y**_2_		**Y**_3_		**Y**_4_	
	**f-value**	**p-value**	**f-value**	**p-value**	**f-value**	**p-value**	**f-value**	**p-value**
Linear vs Mean	105.39	< 0.0001	144.02	< 0.0001	34.06	< 0.0001	148.46	< 0.0001
2 FI vs Linear	0.034	0.8584	1.64	0.2323	9.00	0.0127	1.76	0.2178
Quadratic vs 2 FI	12.71	0.0047	6.07	0.0295	23.09	0.0008	18.21	0.0017

**Table 5:** Model summary statistics of responses to select suitable model to fit data

**Source**		**Linear**			**2 FI**			**Quadratic**		
		**Adj. R** ^2^	**Pred. R** ^2^	**PRESS**	**Adj. R** ^2^	**Pred. R** ^2^	**PRESS**	**Adj. R** ^2^	**Pred. R** ^2^	**PRESS**
**Response**	**Y1**	0.9456	0.9104	**529.8**	0.9398	0.8264	1027.58	**0.9833**	0.9085	**541.08**
	**Y2**	0.9597	0.9382	**2.183 E + 007**	0.9622	0.9346	2.309 E + 007	**0.9822**	0.9165	**2.948 E + 007**
	**Y3**	0.8464	0.6812	132.59	0.9147	0.6934	127.53	**0.9856**	0.9239	**31.67**
	**Y4**	0.9609	0.9338	41.21	0.9637	0.9290	44.22	**0.9925**	0.9630	**23.04**

*Drug content uniformity study of topical bioadhesive gels of Aceclofenac*

All prepared gels were analyzed for aceclofenac content and the content of drug in each prepared TBG ranged from 96% to 104%. The drug content of the prepared TBG was within desired range of 90% to 110% (21).

*Effect of formulation variables on bioadhesion*

From the p-values presented in Table 4, linear model and quadratic model was found to be significant for bioadhesion. Quadratic model was selected on the basis of maximum value of adj. R^2^ and low PRESS value indicating adequate fitting of model (Table 5). Quadratic model was significant with model f-value of 142.32 (p-value < 0.0001). The quadratic equation generated by software is as follows:

Y_1_ = 60.66 + 10.50X_1_ + 28.83X_2_ + 0.50X_1_X_2_ + 0.71X_1_^2^ - 8.29X_2_^2^

**Figure 1:**
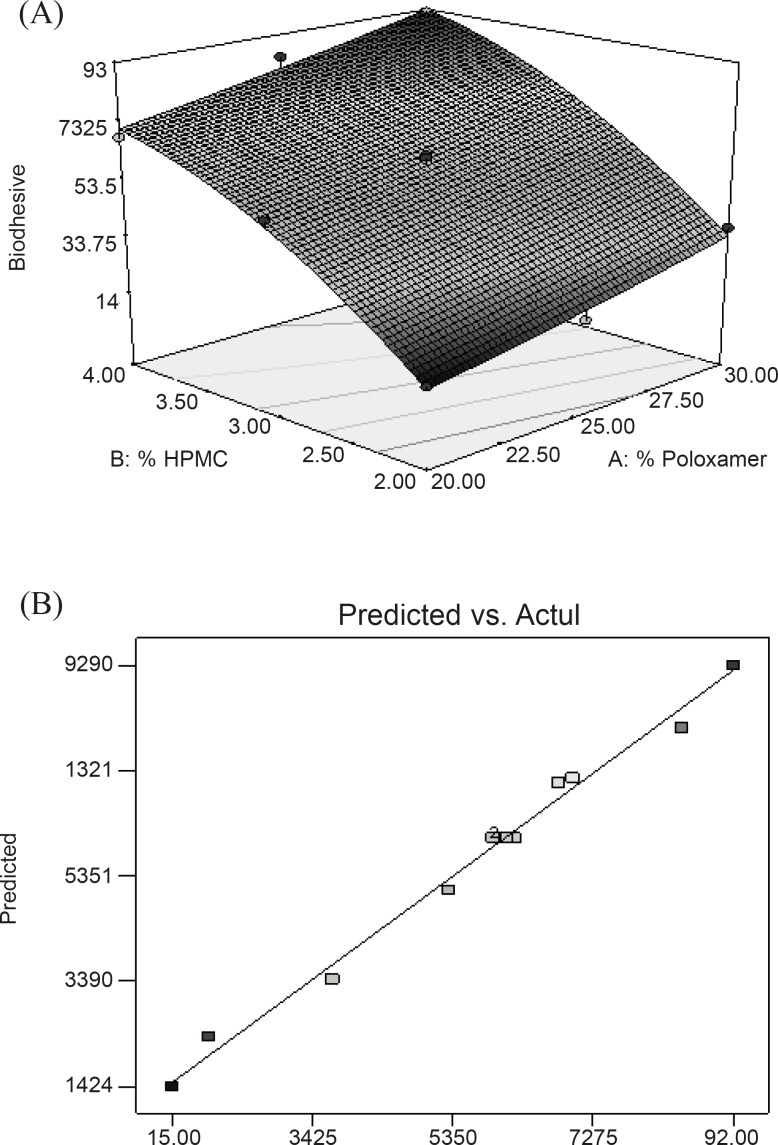
(A) Response surface plot showing the effect of PL-407 and HPMC on Bioadhesion (Y_1_); (B) Linear plot between observed and predicted value of Y_1_

Equation reveals that both factors (X_1_ and X_2_) affect bioadhesion characteristics of gel significantly. Equations also indicated that the effect of the change in HPMC concentration seems to be more pronounced in comparison with that of the change in PL-407 concentration since the coefficient of factor X_2_ has a larger value than that of factor X_1_. The combined effect of factors X_1_ and X_2_ can further be elucidated with the help of response surface plots (Figure 1A ), which demonstrated that Y_1_ varies in a linear fashion with the amount of both the polymers. However, the steeper ascent in the response surface with HPMC (X_2_) – instead of Poloxamer (X_1_) – is clearly discernible from response surface plots, indicating that the effect of HPMC is comparatively more pronounced than that of Poloxamer. From this discussion, one can conclude that the bioadhesion may be changed by appropriate selection of the levels of *X*_1_ and *X*_2_. Figure 1B shows a linear relationship between the observed response values and the predicted values indicating the correctness of the model.

*Effect of formulation variables on rheological properties (consistency index)*

It is essential for any formulation to study its rheological behavior to be used for topical drug delivery applications. It is important for its efficacy in delivering molecules onto or across the skin. Rheological studies of all the gels were done to study the effect of polymer proportion on the viscosity. Consistency index and flow index were calculated for all the batches. Consistency index (CI) was a measure of consistency and equivalent to apparent viscosity at a shear rate of 1 sec^-1^. The flow index (FI) was a measure of the deviation of a system from Newtonian behavior (n = 1). Value of n < 1 indicates pseudoplastic flow or shear thinning system whereas n > 1 indicates dilatant flow or shear thickening system (28). All the gels showed a flow index of less than 1 (data not shown), indicating pseudoplastic flow behavior. Thus, gel becomes thin when applied on the skin and provide better spreadability. Values of consistency index (Y_2_) were summarized in Table 3. Mathematical modeling was applied to result obtained for the consistency index to evaluate the effect of independent variable (HPMC and Poloxamer content in gel) and interaction of two independent variables on rheological properties of gel. From the p-values presented in Table 4, linear contribution and quadratic contribution were found to be significant as p-value is less than 0.05 for both sources. To further fit the data to suitable model, the software was used to analyze the value of adjusted R^2^, predicted R^2^ and Predicted Residual Sum of Square (PRESS). Low PRESS value indicates adequate fitting of quadratic model (31). PRESS value for Y_2_ response (consistency index) showed no significant difference between the linear model and quadratic model (Table 5). The value of Adj. R^2^ of quadratic model was more than that of linear model (Table 5). Moreover, quadratic model was selected by software because of the highest order of polynomial model. Quadratic model was significant with model f-value of 133.53 (p-value < 0.0001). The quadratic equation generated by software is as follows:

Y_2_ = 10396.93 + 1664.6X_1_ + 7353.17X_2 _+ 675.50X_1_X_2_ - 85.76X_1_^2^ + 1432.74X_2_^2^

**Figure 2:**
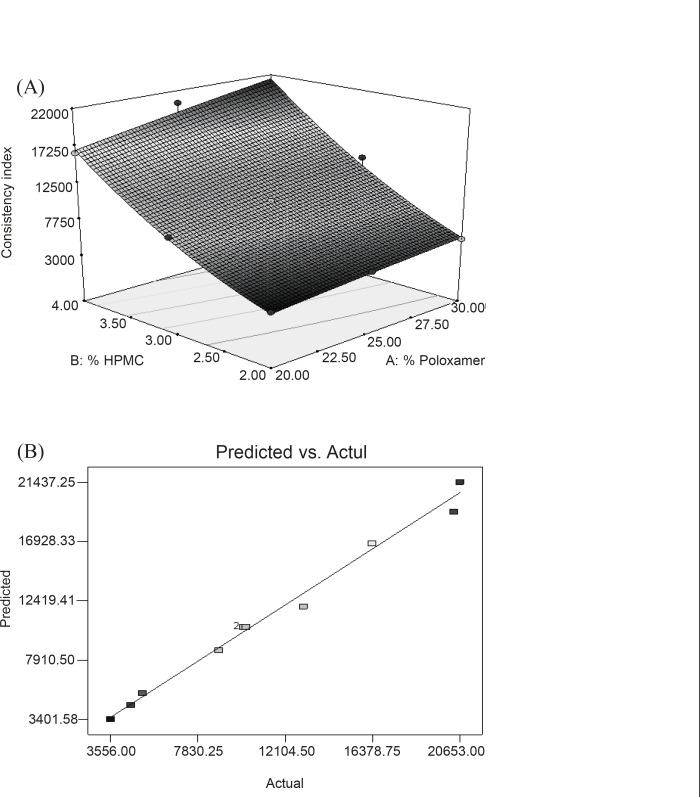
(A) Response surface plot showing the effect of PL-407 and HPMC on Consistency Index (Y_2_) (B) Linear plot between observed and predicted value of Y_2_

In this case, X_1_**, **X_2_ and X_2_^2^ are significant model terms. The equation represents the quantitative effect of factors (X_1_ and X_2_) upon the consistency index (Y_2_ response). Increased concentration of polymers resulted in a stronger gel structure as reflected by equation generated for quadratic model. Equation also reveals that HPMC has more pronounced effect than PL-407 on the consistency index of gel. Surface (Figure 2A) showed a steeper ascent in the response surface with HPMC (X_2_) than with Poloxamer (X_1_). It was in accord with equation generated by software showing pronounced effect of HPMC on consistency index. Model term X_2_^2^ was also found to be significant (Table 6). This can be explained on the fact that HPMC swells in water and forms three disordered dimensional-physical networks. Tightly oriented gel structures are formed within poloxamer micelle pathways by HPMC-molecule entanglement and extensive hydrogen binding (32). Figure 2B represented the observed response value compared with that of predicted values indicating the correctness of model.

**Table 6:** Analysis of variance (ANOVA) table for measured responses

**Model/Model term**	**Y**_1_		**Y**_2_		**Y**_3_		**Y**_4_	
	**f-value**	**p-value**	**f-value**	**p-value**	**f-value**	**p-value**	**f-value**	**p-value**
**Model**	142.32	< 0.0001	133.53	< 0.0001	164.78	< 0.0001	317.22	< 0.0001
**X**_1_	80.33	< 0.0001	31.79	0.0008	412.77	< 0.0001	458.46	< 0.0001
**X**_2_	605.74	< 0.0001	620.23	< 0.0001	311.76	< 0.0001	1082.71	< 0.0001
**X**_1_ **X**_2_	0.12	0.7377	3.49	0.1040	53.19	0.0002	8.47	0.0226
**X**_1_^2^	0.17	0.6945	0.039	0.8494	0.75	0.4159	12.55	0.0094
**X**_2_^2^	23.07	0.0020	10.84	0.0133	34.85	0.0006	34.43	0.0006

Significant p-values (p < 0.05) are in **bold **text

*Effect of formulation variables on cumulative percentage release in 2 h*

For this response, all the models (linear, 2FI and quadratic) are found to be significant with p-value < 0.05 (Table 4). Among all the models, lowest PRESS value was found for quadratic model. Therefore, quadratic model was selected to fit the data of this response (Table 5). Quadratic model was significant with model f-value of 164.78 (p-value < 0.0001). 

The quadratic equation generated by software is as follows:

Y_3_ = 22.56 - 5.87X_1_ - 5.10X_2_ + 2.58X_1_X_2_ + 0.37 X_1_^2^ + 2.51X_2_^2^

**Figure 3:**
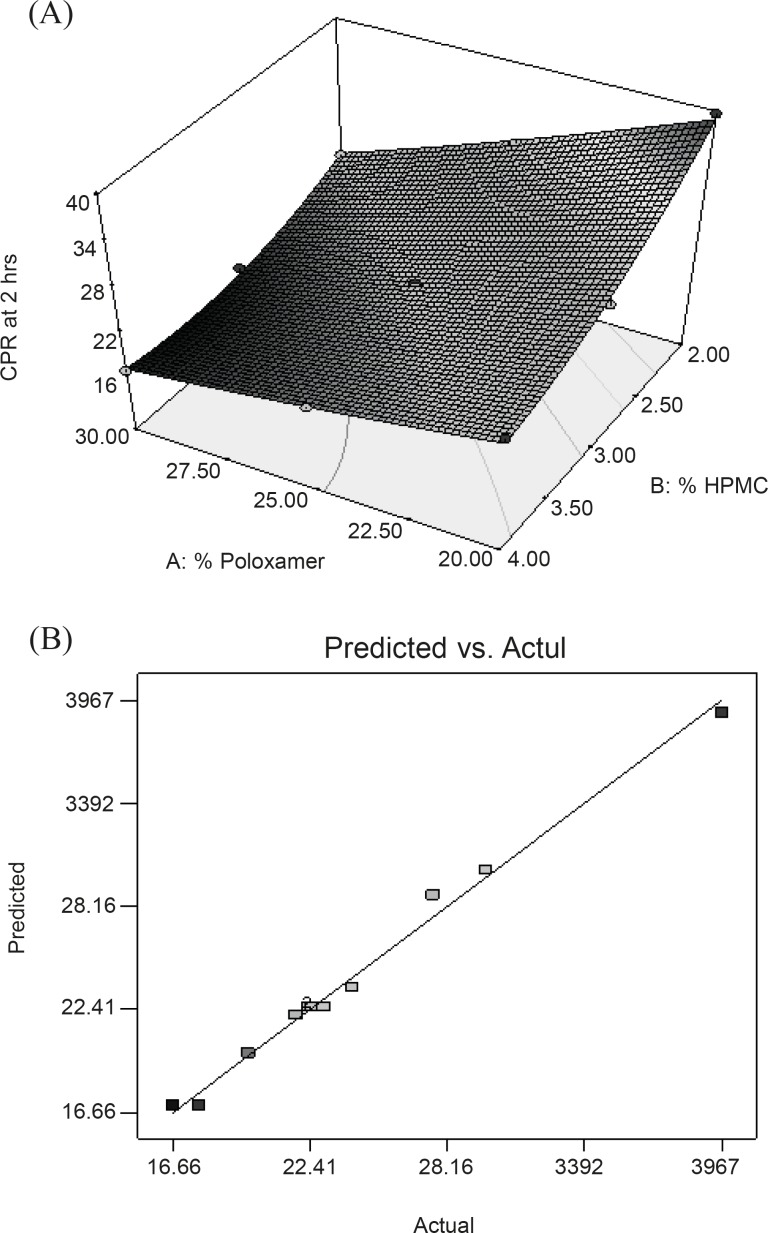
(A) Response surface plot showing the effect of PL-407 and HPMC on cumulative percentage release of drug in 2h (Y_4_); (B) Linear plot between observed and predicted value of Y_3_.

In this case, X_1_, X_2_, X_1_X_2_ and X_2_^2^ were found to be significant model terms (Table 6)**. **Equation reveals that both factors have antagonistic effect on the drug release. Figure 3A displayed a non-linear relationship for CPR in 2 h at high levels of the polymers. This can be attributed to the occurrence of potential interaction between the two polymers at the corresponding factor levels, construing that each polymer tends to modify the effect of the other one toward the drug release. Model term X_1_X_2_ was found to be significant which indicating the potential effect of this interaction on the drug release in 2 h. Figure 3A also displayed the non-linearity of the response at high concentration of HPMC supported by the model term X_2_^2^ which is found to be significant for the response. This could be due to the rigid gel structure formation due to the interaction between HPMC and PL-407 as already explained in consistency index. Figure 3B represented the observed response values compared with that of predicted values indicating the correctness of the model.

*Effect of formulation variables on cumulative percentage release in 8 h*

From the p-values presented in Table 4, linear contribution and quadratic contribution were found to be significant since p-value is less than 0.05 for both sources. In this case, A, B, AB, A^2^ and B^2^ are significant model terms. PRESS value for quadratic model (23.04) was found lower than that of linear model (41.^21^) as indicating in Table 5. Therefore, quadratic model was selected to fit the data of this response. Quadratic model was significant with model f-value of 317.22 (p-value < 0.0001). The quadratic equation generated by software is as follows:

Y_4_ = 50.64 - 5.47X_1_ - 8.40X_2_ + 0.91X_1_X_2_ - 1.33X_1_^2^ + 2.21X_2_^2^

**Figure 4:**
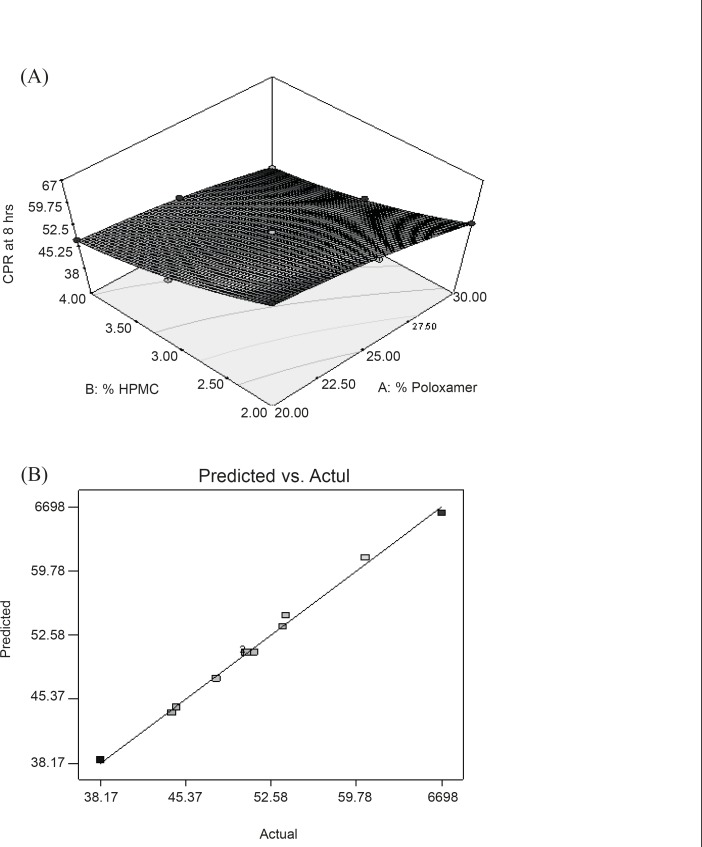
(A) Response surface plot showing the effect of PL-407 and HPMC on cumulative percentage release of drug in 8h (Y_4_); (B) Linear plot between observed and predicted value of Y_4_.

In this case, all the model terms (X_1_**, **X_2_, X_1_X_2_, X_1_^2^ and X_2_^2^) were found to be significant (Table 6)**. **The equation reveals that both factors have antagonistic effect on the drug release. CPR of the drug in 8 h with highest polymer content (30% Poloxamer and 4% HPMC) was found to be lowest. However, in this equation, it was clearly indicating that the retarding effect of HPMC was more prominent than PL-407. Coefficient of interactions shown in above equation was also significant which confirms the formation of rigid gel structure of PL-407 with HPMC. At high concentration of HPMC and PL-407, a very thick gel (highest consistency index value) was formed which provide a very slow release of drug. Figure 4A represented the response surface indicating the more pronounced effect of HPMC than PL-407 on Y_4_. Figure 4B represented the observed response value compared with that of predicted values indicating the correctness of model.

## Conclusion

The present study concludes that topical bioadhesive gel of aceclofenac can be formulated by using combination of Poloxamer 407 and HPMC employing the response surface approach. The effect of formulation variables on the product characteristics can be easily predicted and precisely interpreted by using a 3-level factorial experimental design and generated quadratic mathematical equations. On the basis of product characteristics such as bioadhesive strength, consistency index and *in-vitro *release, it can be concluded that the best batch of topical bioadhesive gel of Aceclofenac would be with 20% PL-407 and 3% HPMC.
